# Efficacy and safety of immune checkpoint inhibitors for locoregionally advanced, recurrent and metastatic nasopharyngeal carcinoma: a systematic review of phase III randomised controlled trials

**DOI:** 10.1007/s00210-025-04572-3

**Published:** 2025-09-15

**Authors:** Sivananthan Manoharan, Lee Ying Ying

**Affiliations:** 1 Molecular Pathology Unit, Cancer Research Centre, Institute for Medical Research, National Institutes of Health, Ministry of Health Malaysia, Setia Alam, 40170 Shah Alam, Selangor Malaysia; 2 Asia Metropolitan University, Bandar Baru Seri Alam, 81750 Johor Bahru, Johor Malaysia

**Keywords:** Immune checkpoint inhibitors (ICIs), Nasopharyngeal carcinoma (NPC), Randomised controlled trials (RCTs), Systematic review

## Abstract

Immune checkpoint inhibitors (ICIs) are an emerging treatment option for cancer. Many randomised controlled trials (RCTs) using ICIs have been conducted on nasopharyngeal carcinoma (NPC) patients, some of which are phase III trials of locoregionally advanced and recurrent/metastatic NPCs. We performed a systematic review to qualitatively assess the efficacy and safety of ICIs in NPC patients. PubMed/MEDLINE, ScienceDirect, Google Scholar, Google, preprint servers, and references were thoroughly searched using Boolean search string (except for references) from inception through April 28, 2025. Full articles and conference abstract that met the eligibility criteria, such as reported studied population, intervention, comparison and outcome, were included. The quality of included studies was assessed with the Cochrane risk of bias (RoB) version 1.0 tool. Meta-analysis was not performed due to data heterogeneity, mainly from methodological variations. Seven phase III trials were shortlisted and tabulated. Three studies described on locoregionally advanced NPC (LANPC), with one article showing moderate RoB and two articles showing high RoB, while three out of four studies on recurrent/metastatic NPC had low RoB. Studies with high RoB provide low certainty/reliability, while those with low RoB offer higher reliability. Each study was unique in ICI usage and study design, including disease stage, adjuvant versus combination therapy, and participant selection criteria. Three high RoB studies, with two studies belonging to LANPC, reported negative effects (p > 0.05) on overall survival (OS). Two low RoB recurrent/metastatic studies reported positive outcome. Progression-free survival (PFS) was met (p < 0.01) in three low RoB recurrent/metastatic NPC studies. PFS [Hazard ratio-HR 0.52 (95% Confidence interval (CI): 0.37–0.73); p < 0.001] and OS [HR 0.63 (95% CI: 0.45–0.89); p < 0.01] were met in 1 low RoB recurrent/metastatic study (toripalimab). Overall, grade ≥ 3 adverse events, including immune-related ones (with rashes being the most common), were manageable. Based on our systematic review, combining ICIs with standard chemotherapy is probably a promising approach for PFS in recurrent/metastasic NPC patients. However, due to high RoB it is inconclusive for LANPC, and high-quality studies are needed.

## Introduction

Head and neck cancers are diagnosed in more than 550,000 patients and result in 380,000 mortalities worldwide annually (Jicman et al. 2022). Nasopharyngeal carcinoma (NPC) is a type of head and neck cancer originating from epithelial tissue located in the nasopharynx region. The Epstein-Barr virus (EBV) infection further complicates this cancer. NPC is commonly diagnosed in Southeast Asia, affecting more males than females (Jicman et al. 2022; Hsu et al. 2025). NPC is highly malignant and is susceptible to lymph node metastasis and distant metastasis with no apparent symptoms at the early stage of the disease (Suryani et al. 2024). NPC is sensitive to radiotherapy (Chen and Hu 2015), but approximately 15–58% of the patients will develop a recurrence, and Many of them will experience distant metastases. This group of patients usually experiences a poor prognosis with a median overall survival of only 20 months (Suryani et al. 2024). Due to recurrence and metastasis, cisplatin + gemcitabine chemotherapy intervention is often required (Zhang et al. 2019). Despite these treatment approaches, the OS of NPC patients is still below satisfactory. The development of radiotherapy- or chemotherapy-related resistance, recurrence, or distant metastasis after radiotherapy and treatment-related high toxicity in advanced-stage NPC patients contributes to the situation (Siak et al. 2023). The asymptomatic nature of early-stage NPC often leads to advanced-stage diagnosis, further complicated by limited treatment options and increased risk of disease recurrence and distant metastasis (Siak et al. 2023).

Immunotherapy, mainly immune checkpoint inhibitors (ICIs), for instance, anti-programmed death 1/programmed death-ligand 1 (PD-1/PD-L1) treatment, has appeared as an important treatment approach for solid tumours, including NPC, where this cancer has high PD-L1 expression (Liu et al. 2024a). Immunotherapy regulates the immune reaction inside the tumour microenvironment to inhibit or eradicate the tumour cells. The presence of EBV infection, neoantigens, highly tumor-infiltrating lymphocytes in the tumour microenvironment, and up to 90% expression of PD-L1 in cancer cells are the unique features of NPC which make immunotherapy a potential treatment for NPC (Cai et al. 2024; Adkins and Haddad 2022; Yiu 2018; Zhou et al. 2017; Yang et al. 2023a; Ma et al. 2020). Through RCTs, ICIs have been tested in LANPC and recurrent/metastatic NPC. In NPC, combinations of standard treatment with ICIs are actively ongoing. We conducted a systematic review to evaluate the efficacy and safety of ICIs in NPC patients. Despite the availability of phase I, I/II, and II studies, only phase III RCTs are included in this systematic review. Phase III studies are more robust and multicentred, have included more patients in the studied arms, and have the potential to influence policy. Meta-analysis was not conducted due to the heterogeneous nature of the extracted data. For example, none of the included studies have similarities in using ICIs except for two. Although these two studies used the same ICI (tislelizumab), it was tested in 2 different settings: LANPC and recurrent/metastasis. Despite all ICIs in the current systematic review targeting PD-L1, pooling the outcomes from different ICIs, treatment settings, and interventions in a meta-analysis could lead to a greater RoB in the generated report and could not be used to guide and may mislead clinicians and policymakers.

## Methodology

In this systematic review, the Preferred Reporting Items for Systematic Reviews and Meta-Analyses (PRISMA) guidelines were followed accordingly.

### Research questions

1. Are the ICIs effective when added to the existing standard treatment?
2. Are the ICIs causing more adverse effects when added to the existing standard treatment?

#### Search strategies, article inclusion criteria, data recording process, and risk of bias assessment

PubMed/MEDLINE, ScienceDirect, Google Scholar, Google, preprints, and references lists were searched systematically using Boolean search string designated as “Immune checkpoint inhibitors” AND “nasopharyngeal carcinoma” OR “Immune checkpoint inhibitors” AND “nasopharyngeal carcinoma” AND “randomised controlled trials” OR “PD-1/PD-L1 inhibitor” AND “nasopharyngeal carcinoma” AND “randomised controlled trials” OR “Immune checkpoint inhibitors for head and neck cancers” AND “randomised controlled trials” OR “CTLA-4 inhibitor” AND “nasopharyngeal carcinoma” AND “randomised controlled trials”. The Boolean search string for example “Immune checkpoint inhibitors” AND “nasopharyngeal carcinoma” AND “randomised controlled trials” was used to search in each database except for reference list where Manual search was performed. The search year was between inception and February 26, 2025. An additional article search was carried out from the 21 st to the 28th of April 2025, and no new article was identified. No filters were used during the article search. The article inclusion criteria were:

1. Full-length articles and conference abstracts.
2. Inclusion of NPC patients diagnosed with LANPC or recurrent/metastatic disease.
3. The use of ICI was documented.
4. The study was properly designed with appropriate controls.
5. The study was strictly limited to phase III RCTs.
6. The article states clinical efficacy and side effects.
7. Only articles written in English are included.

The data extraction and recording procedures, including details such as ICI and NPC types, study period, study site, sample size, median follow-up time, median age, interventions in control and studied arms, and outcomes with and without p-values, were fully conducted independently by two authors (SM & LYY). Disagreements were resolved through discussion, and a third reviewer was not required. The conference abstracts were only included if the abstracts described a phase III study published in a reputable journal, like the Journal of Clinical Oncology. In the current clinical context, a reputable journal is defined as one with a double-digit impact factor and as one of the major sources of reference for clinicians and policymakers. This criterion ensures inclusivity and provides comprehensive information about ICIs for NPC. There have been cases where including conference papers has changed the estimated treatment effect, not just the estimate's precision. Instead of making arbitrary choices about whether to include conference abstracts in systematic reviews, the authors suggest that those conducting the reviews consider all available evidence to guide their decisions (Scherer and Saldanha 2019). Since no meta-analysis was conducted in the current work, including a conference abstract primarily aims to qualitatively assess the study's quality and key outcomes. The quality of abstracts was evaluated with the same criteria used for full-length articles. The Cochrane RoB version 1.0 tool was employed to assess risk of bias. This version of RoB tool includes 7 criteria known as (1) random sequence generation, (2) allocation concealment, (3) blinding of participants and personnel, (4) blinding of outcome assessment, (5) incomplete outcome data, (6) selective reporting and (7) other bias. The methods used in this systematic review were adapted from the authors’ published systematic review and meta-analysis (Manoharan and Ying 2024). Meta-analysis was not performed due to methodological differences, such as each study reporting different ICIs and varying stages of NPC, like LANPC and recurrent/metastatic disease. For example, despite two studies reporting on tislelizumab, they were not pooled for meta-analysis because one focused on LANPC and the other on recurrent/metastatic NPC. During the revision phase after the initial peer review, a full-length article published in March 2025 (Liang et al. 2025) was identified. This latest article is a full version of an included conference abstract (Ma et al. 2024), leading to an update of Table 1 with the most recent information.

**Table 1:** Characteristics of the included studies

Study	Study design & phase & ICI	NPC type	Period of study	Study site & median follow-up	Population T vs C & median age (T vs C)	Intervention	Outcomes with p-values reported in the literatures T vs C (in % unless stated otherwise)	Outcomes without p-values reported in the literatures T vs C (in % unless stated otherwise) with common adverse event (in %)	Cochrane RoB version 1.0
Mai et al. (2024) (ASCO Meeting abstract)	Full RCT & phase III trial & **tislelizumab**	LANPC	June 22-May 23	Multicentre & NA	**450** (223 vs 227) & NA	200 mg tislelizumab or placebo + Cis & Gem every 3 weeks for 3 cycles. Then CCRT & adjuvant tislelizumab/placebo every 3 weeks for up to 8 cycles	30.5 vs 16.7 (**CRR**) (p < 0.001)	**Grade ≥ 3 AE** 40.6 vs 39.3 **SAEs** 2.3 vs 1.3	**Moderate** (Caution is needed due to abstract only publication)
Liang et al. (2025)	Full RCT & phase III trial & **camrelizumab**	LANPC	Aug 18-Nov 21	Multicentre & 39 months	**450** (226 vs 224) & 46 vs 46	Adjuvant 200 mg camrelizumab every 3 weeks for 12 cycles (camrelizumab treatment arm) or observation (standard treatment arm) (Note: Adjuvant setting)	86.9 vs 77.3 (**EFS**) (p < 0.05) 92.4 vs 84.5 **(DMFS)** (p < 0.05) 92.8 vs 87 **(LRFS)** (p < 0.05) 96.4 vs 92.9 (**OS**) (p > 0.05)	**Grade 3 & 4 AE** 11.2 vs 3.2 Leukopenia (4.9 vs 1.4)	**High** (due to open labelled study)
Liu et al. (2024b)	Full RCT & phase III trial & **sintilimab**	LANPC	Dec 18-March 20	Multicentre & 41.9 months	**425** (210 vs 215) & 46 vs 46	Cis & Gem + CCRT (standard treatment arm) with or without 200 mg sintilimab every 3 weeks for 12 cycles	86 vs 76 **(EFS)** (p < 0.05) 90 vs 83 **(DMFS)** (p < 0.05) 93 vs 86 **(LRFS)** (p < 0.05) 92 vs 92 **(OS)** (p > 0.05)	**Grade 3 & 4 AE** 74 vs 65 Leukopenia (27 vs 22) **Immune AE** 10 vs NA Rash (4 vs NA)	**High** (due to open labelled study)
Chan et al. (2023)	Full RCT & phase III trial & **pembrolizumab**	Recurrent or metastatic	May 16-May 18	Multicentre& 45.1 months	**233** (117 vs 116) & 51 vs 53	200 mg pembrolizumab every 3 weeks or capecitabine orally 2 times daily from day 1–14 per 3-week cycle, Gem on day 1 & 8 per 3-week cycle, docetaxel day 1 per 3-week cycle. Pembrolizumab continued up to 35 cycles and chemotherapy > 35 cycles if it is tolerated (Note: platinum pre-treated patients)	17.2 vs 15.3 months **(OS)** (p > 0.05)	4.1 vs 5.5 months **(PFS)** 12 vs 13.1 months** (DOR)** 2.8 vs 1.5 months** (TTR)** 7.7 vs 10.3 **(CRR)** 13.7 vs 12.9 **(PRR)** 37.6 vs 21.6 **(PDR)** 29.1 vs 40.5** (SDR)** **Grade ≥ 3 AE** 10.3 vs 43.8	**High** (due to open labelled study)
Mai et al. (2023)	Full RCT & phase III trial & **toripalimab**	Recurrent or metastatic	Nov 18-Oct 19	Multicentre & 39 months	**289** (146 vs 143) & 46 vs 51	240 mg toripalimab or placebo with Cis & Gem for 6 cycles followed by maintenance with toripalimab/placebo	21.4 vs 8.2 months **(PFS)** (p < 0.001) 78.8 vs 67.1 **(ORR)** (p < 0.05) NR vs 33.7 months **(OS)** (p < 0.01)	18 vs 6 months **(DOR)** 26.7 vs 13.3 **(CRR)** 52.1 vs 53.8 **(PRR)** 9.6 vs 13.3 **(SDR)** 88.4 vs 80.4** (DCR)** 3.4 vs 5.6** (PDR)** **Grade ≥ 3 AE** 89.7 vs 90.2 Leukopenia (61.6 vs 58.7) **Immune AE** 9.6 vs 1.4 Rash (3.4 vs 0.7)	**Low**
Yang et al. (2021)	Full RCT & phase III trial & **camrelizumab**	Recurrent or metastatic	Nov 18-Nov 19	Multicentre & 15.6 months	**263** (134 vs 129) & 52 vs 49	200 mg camrelizumab or placebo plus Cis & Gem every 3 weeks for 4–6 cycles then maintenance with camrelizumab or placebo	9.7 vs 6.9 months **(PFS)** (p < 0·001)	8.5 vs 5.6 months **(DOR)** 1 vs 3** (PDR)** 9 vs 14** (SDR)** 87.3 vs 80.6 **(ORR)** 5 vas 3 **(CRR)** 82 vs 78 **(PRR)** 96.3 vs 94.6** (DCR)** **Grade ≥ 3 AE** 94 vs 91 **SAEs** 44 vs 37	**Low**
Yang et al. (2023b)	Full RCT & phase III trial & **tislelizumab**	Recurrent or metastatic	April 19-Sept 20	Multicentre & 15.5 months	**263** (131 vs 132) & 50 vs 50	200 mg tislelizumab or placebo with Cis & Gem for 4–6 cycles	9.6 vs 7.4 months **(PFS)** (p < 0.001)	8.5 vs 6.1 months **(DOR)** NR vs 23 months** (OS)** 16 vs 6.8 **(CRR)** 53.4 vs 48.5 **(PRR)** 69.5 vs 55.3 **(ORR)** 14.5 vs 25.8** (SDR)** 3.1 vs 10.6** (PDR)** 89.3 vs 84.8 **(DCR)** 77.1 vs 64.4** (CBR)** **Grade ≥ 3 AE** 80.9 vs 81.8 **Immune AE** 2.3 vs NA Rash (1.5 vs NA)	**Low**

*CRR* Complete response rate, *AE* Adverse events, *SAE* Serious adverse event, *EFS* Event free survival, *DMFS* Distance metastasis free survival, *LRFS* Locoregional recurrence free survival, *OS* Overall survival, *NA* Not applicable, *Cis* Cisplatin, *Gem* Gemcitabine, *PFS*, Progression free survival, *PRR* Partial response rate, *PDR* Progressive disease rate, *DOR* Duration of response, *TTR* Time to response, *SDR* Stable disease rate, *ORR* Objective response rate [CRR + PRR], *DCR* Disease control rate, *CBR* Clinical benefit rate, *NR* Not reached, *ASCO* American Society of Clinical Oncology, *T* Treatment, *C* Control

## Results

**Fig. 1:**
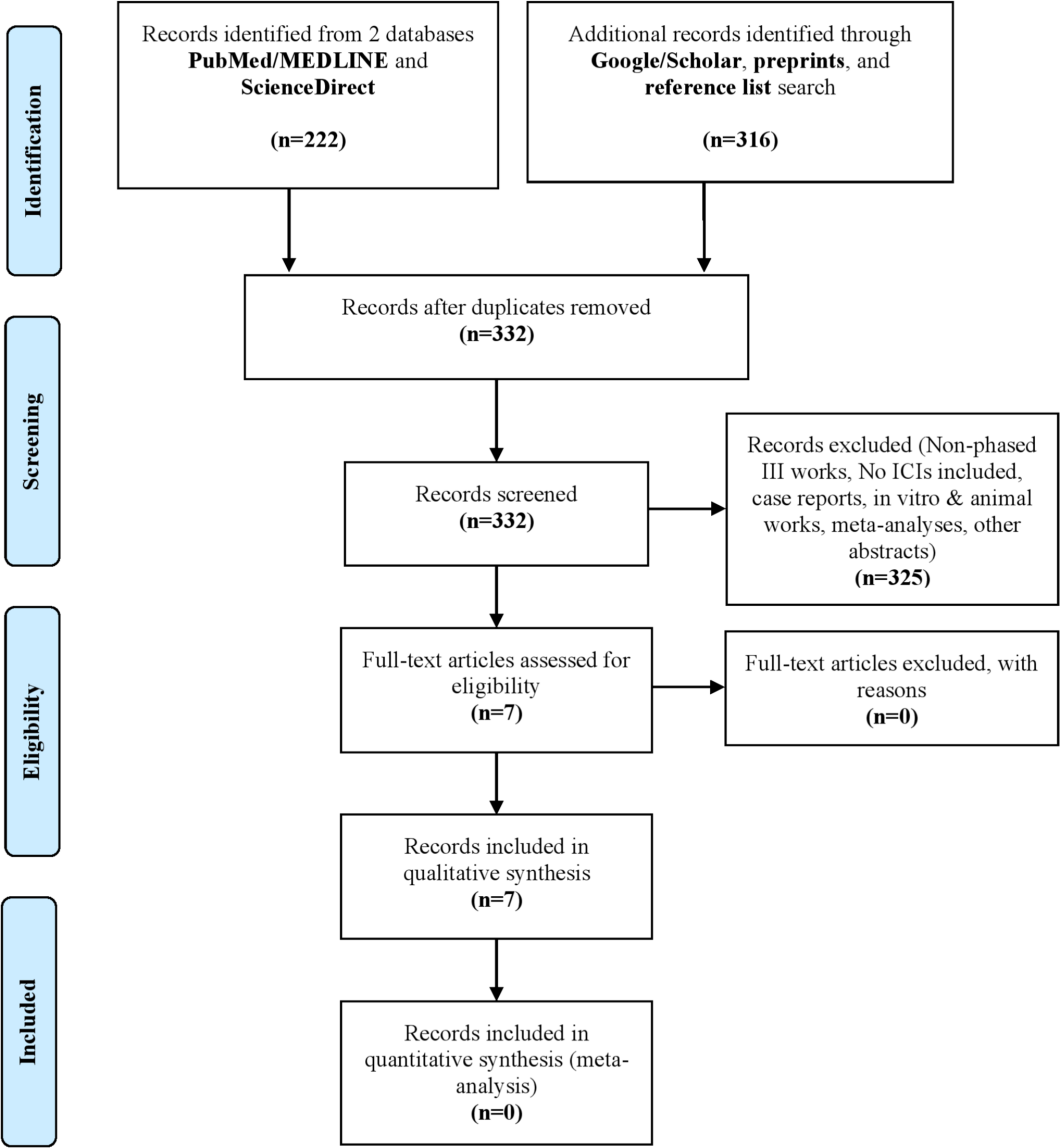
PRISMA flow chart of articles inclusion and exclusion (Moher et al. 2009)

**Fig. 2:**
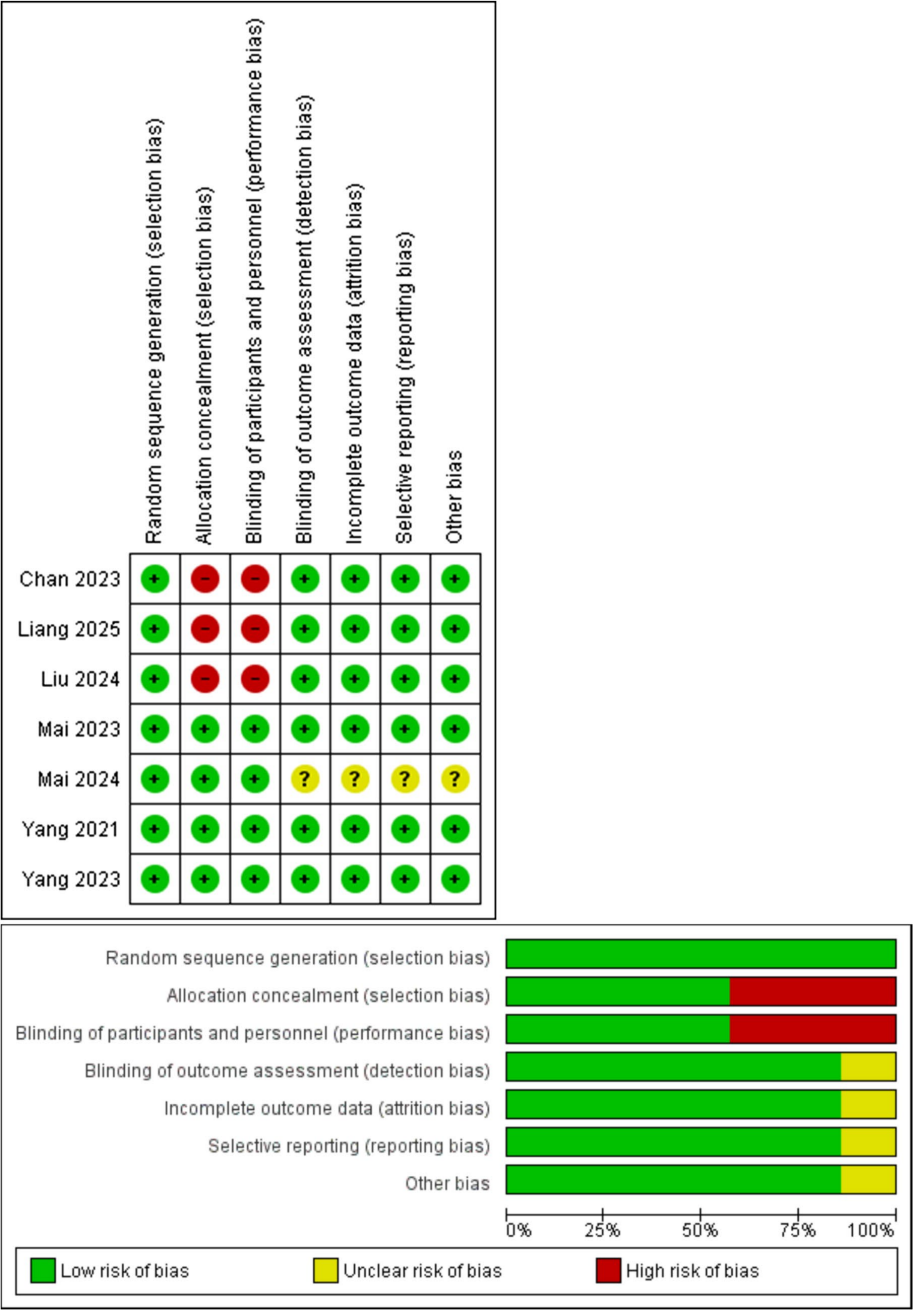
The RoB 1.0 analysis generated from Review Manager 5.4.1 (Review Manager (RevMan), [Computer Program]. Version 5.4, The Cochrane Collaboration 2020)

Out of 538 literatures in Fig. 1, the characteristics of 7 articles that meet the inclusion criteria are detailed in Table 1 for further discussion. Based on Table 1, three articles are classified as LANPC, while the other four are classified as recurrent/metastatic. One out of the seven articles (1/7) is a conference abstract. Most studies were conducted by Chinese authors, which is understandable Because NPC is endemic in East Asia, especially China and Southeast Asia. The median age of patients in both the ICIs and control groups ranged from 46 to 52 and 46 to 53 years, respectively. The shortest follow-up duration was 15.5 months (recurrent/metastasis), and the longest was 45.1 months (recurrent/metastasis). Regarding the risk of bias (RoB), for recurrent/metastatic NPC, three studies are categorised as low RoB and one as high RoB, as outlined in Table 1 and shown in Fig. 2. The remaining three studies involve LANPC, with two classified as high RoB and one as moderate RoB. According to Fig. 2, all studies employed randomisation, which is an important step for RCTs. However, three studies did not perform the allocation concealment or blinding of participants or investigators. Except for Mai et al. (2024), which is a conference abstract, the other studies met the remaining four criteria for assessing RoB. There is no specific tool for evaluating RoB in abstracts. Since conference abstracts are concise summaries of key phase III outcomes, they reveal limited information regarding RoB. We did not classify them as high RoB because, although the authors did not specify this, we could not assume these aspects were not addressed. Therefore, we assessed these criteria as an unclear RoB. Inclusion of the conference abstract, which reports on tislelizumab would provide a clearer understanding of the efficacy and safety of ICIs for NPC.

All studies were conducted across multiple sites. Table 1 reports three ICIs for LANPC, known as tislelizumab, camrelizumab, and sintilimab. Each LANPC study had a unique design. Besides differences in the ICIs used, Mai et al. (2024) implemented a combination of ICI and standard chemotherapy for 3 cycles, followed by CCRT and adjuvant ICI or placebo for up to 8 cycles. Liang et al. (2025) and Liu et al. (2024b) focused on adjuvant and combination approaches, respectively, for 12 cycles. In recurrent/metastatic studies, all used a combination strategy (ICI + chemotherapy). While all recurrent/metastatic studies used cisplatin + gemcitabine for up to 6 cycles, only Chan et al. (Chan et al. 2023) used capecitabine/docetaxel for 35 cycles if tolerated. Due to differences in study designs, participant criteria, and high RoB, the outcomes from Chan et al. (Chan et al. 2023) must be analysed individually. Tislelizumab and camrelizumab were used in both LANPC and recurrent/metastatic NPC studies. Despite this, data cannot be pooled because of the disease differences (LANPC versus recurrent/metastatic) and study settings, especially for camrelizumab (adjuvant versus combination). The other three recurrent/metastatic studies were not pooled due to differences in the median follow-up time and ICIs used, even though the disease type is similar. Among five studies [LANPC (2 studies) & recurrent/metastatic (3 studies)] reporting OS, only Mai et al. (Mai et al. 2023) and Yang et al. (Yang et al. 2023b) from recurrent/metastatic studies reported positive results. Both studies had low RoB. Unlike toripalimab and tislelizumab from the recurrent/metastatic group, pembrolizumab, sintilimab, and camrelizumab did not show significant effects on OS (p > 0.05). Although the data HR = 0.60 [95% CI: 0.35–1.01] from Yang et al. (Yang et al. 2023b)/tislelizumab suggests a positive trend for OS, a longer follow-up would provide a clearer picture. The reported median follow-up was 15 months. OS was statistically significant (p < 0.01) in Mai et al. (Mai et al. 2023)/toripalimab study with 39 months of median follow-up. Most studies showed manageable adverse events (grade ≥ 3), except for camrelizumab in LANPC, where adverse events occurred in 11.2% compared to 3.2%, favouring the control. Leukopenia (4.9% vs. 1.4%) was reported as the most common grade ≥ 3 adverse event. The grade ≥ 3 side effects for camrelizumab in the recurrent/metastasis study are also Manageable. Regarding immune-related adverse events, toripalimab had rates of 9.6% vs. 1.4%, favouring control. The commonly reported immune-related adverse event was rash (3.4% vs. 0.7%).

## Discussion

In current systematic review, we qualitatively analyse the efficacy and safety of ICIs reported in Phase III RCT studies for LANPC and recurrent/metastatic NPC. The included articles fairly represent both high- and low-RoB studies. It is crucial to interpret the data based on the nature of the disease (LANPC or recurrent/metastasis) and the RoB. For example, disease characteristics differ between LANPC and recurrent/metastasis. Pooling these articles for interpretation is not recommended. Low RoB recurrent/metastatic RCTs are more reliable compared to LANPC studies, where none of the LANPC articles had low RoB. The high RoB in LANPC mainly results from the open-label design of the studies, except for one study, which is a conference abstract. This performance bias can reduce confidence in the overall results (Manoharan and Ying 2022). Conducting RCTs is complex and resource-intensive. The single- or double-blinding strategies used in large sample size powered phase III RCTs are rigorous. Open-label RCTs are more prone to high RoB because not only the drug administrator but also the patients are aware of the drug name (unless coded) and the trial arm(s). Patients (or their relatives) may look up drug information online, which could influence reporting and documentation. For example, patients in the trial arm(s) might exaggerate side effects. These factors can impact the overall validity of the conclusions (Manoharan 2024) (Phillips et al. 2022). Attention should also be given to combining studies with a high RoB, as their results are unlikely to accurately reflect the true treatment effect. This remains an important consideration even in the absence of clinical or statistical heterogeneity (Lensen 2023). According to the Cochrane Handbook, it is recommended to address the RoB in the meta-analysis, and the primary analysis should focus on studies with a low RoB (Lensen 2023; Higgins et al. 2022). Careful assessments are required on whether a meta-analysis is suitable rather than doing it by default. This involves asking if the studies are similar enough in design and execution, if there is a substantial RoB, and if any statistical heterogeneity has been properly considered (Lensen 2023). People over the age of 65 account for approximately 50% of all cancer cases (Herck et al. 2021), but the median age range in the included articles was much younger. There is a possibility that sponsors and regulatory agencies are more focused on the younger population (Parks et al. 2021), as publicity from successful results tends to be more prominent in this group. Younger participants are significantly less likely to have comorbidities that could lead to higher risks for adverse outcomes. Currently, there is no penalty for excluding older adults, which means there is no incentive for sponsors to encourage their inclusion (Parks et al. 2021). Conversely, patients who are younger (18–49 years) and middle-aged (50–64 years) are more likely to receive chemotherapy compared to those who are older (65–85 years) (Jiang et al. 2020), possibly explaining why older patients are under-represented in RCTs. Yu et al. (Yu et al. 2024) noted that the sample sizes of the recurrent/metastatic trials with low RoB were relatively small because the morbidity rate of NPC is low worldwide. This means individual trials comparing chemotherapy to ICI plus chemotherapy in a first-line setting lacked enough power to determine if there were differences in treatment effectiveness among various patient subgroups (Yu et al. 2024). The authors combined data from different studies and performed a meta-analysis with a larger sample size (Yu et al. 2024). For example, Yu et al. (Yu et al. 2024) found that PFS favoured the ICI + standard chemotherapy group compared to chemotherapy alone with HR = 0.52 [(95% CI: 0.43–0.63); p < 0.001]. This result aligns with those three individual studies, all of which had p < 0.001 (Table 1). This shows that whether pooled or not, the outcome is the same for PFS due to the homogeneity of the individual studies. It is important to note that despite only having a moderate sample size, the US Food & Drug Administration (USFDA) approved toripalimab plus standard chemotherapy for NPC on October 27, 2023 (FDA approves toripalimab-tpzi for nasopharyngeal carcinoma 2023). It is known that trials for rare diseases tend to have smaller sample sizes than those for common diseases (Hee et al. 2017). Recent FDA approvals show that Many clinical studies for cancer indications involved fewer than 200 participants (Ladanie et al. 2020; Beca et al. 2021). While pooling sample sizes can be done, it is not always beneficial because each trial used different ICIs. Additionally, the median follow-up times varied. The significance of post-trial follow-up in large RCTs cannot be overstated. It is essential not only for assessing the long-term effects of an intervention but also for understanding the safety profile and potential risks that may not be evident during the relatively short in-trial period (Llewellyn-Bennett et al. 2016). In chapter 6 (Sect. 6.2.4), Cochrane handbooks and manuals mentioned “Define several different outcomes, based on different periods of follow-up, and plan separate analyses. For example, time frames might be defined to reflect short-term, medium-term, and long-term follow-up” (Higgins et al. 2024). Therefore, in current systematic-review, each study must be analysed separately due to several reasons mentioned above. When comparing three low-RoB recurrent/metastatic studies, toripalimab stands out as an interesting immune checkpoint inhibitor. The significant difference in PFS between toripalimab and control arms likely results from their longer follow-up periods. PFS, a common primary endpoint in cancer trials, offers advantages such as lower costs, quicker trial completion, and fewer participants needed compared to OS (Fallowfield and Fleissig 2011). Compared to low RoB recurrent/metastatic articles, the sole high RoB recurrent/metastatic study’s outcome could be positive if it had a similar design. Based on current evidence, including standard chemotherapy is important for studying ICIs'effects in NPC. This suggests new research avenues, such as exploring pembrolizumab combined with standard chemotherapy. Based on the study period listed in Table 1, the COVID-19 pandemic likely impacted several trials. Cancer drug discovery trials are especially vulnerable to follow-up losses due to the need for frequent onsite visits. During the pandemic, treatment delays can lead to early withdrawals (Huang et al. 2022). The benefits of treatment, such as tumour shrinkage or stabilization, must be balanced against potential harms from drug therapy (Fallowfield and Fleissig 2011). The addition of ICI to standard chemotherapy resulted in a higher incidence of adverse events compared to using chemotherapy alone. Perdigoto et al. (Perdigoto et al. 2021) noted that immune-related adverse events are common across all cancer types with ICI use. These events differ from traditional treatments; they often have delayed onset and prolonged duration and can affect any organ or system. While usually manageable and reversible, some adverse effects may be severe and cause lasting health issues (Yin et al. 2023). For neurological, haematological, and cardiac toxicities, treatment should be stopped immediately. For grade III immune-related adverse events, if ICI therapy continues, high-dose corticosteroids should be started and tapered over 4 to 6 weeks. If symptoms persist for 2–3 days, immunosuppressants like infliximab may be used. ICI therapy should be permanently discontinued if grade IV toxicity occurs, except for endocrine disorders, which can be managed with hormone replacement therapy (Yin et al. 2023). However, managing immune-related adverse events often involves immunosuppression, which can weaken anticancer immune responses. Ongoing issues include unclear mechanisms, lack of predictive biomarkers, methods for early detection, and development of personalized treatment strategies (Yin et al. 2023). Some adverse events are difficult to detect due to vague symptoms, especially rare ones. As ICI use increases, further clinical research is needed to better understand and manage immune-related toxicities, including biomarker development and education for physicians and patients (Casagrande et al. 2024). The current systematic review has some limitations. First, a meta-analysis was not conducted even though two studies, Cai et al. (Cai et al. 2025) and Yu et al. (Yu et al. 2024), performed meta-analyses. Our study differs from these two articles primarily because of the methodological differences in the included articles, as mentioned above. Second, the moderate to high risk of bias in LANPC articles Makes it difficult for us to draw conclusions. Third, most of the studies were conducted by Chinese authors, with some research sites and centres located outside China, but NPC cases from Africa or Many Western countries were not included. Only cases from Canada and United States of America were included in 1 study. Fourth, a conference abstract (1/7) published in a high-impact-factor journal was used, and the justifications have been discussed above. In conclusion, combining ICI with standard chemotherapy appears to be a promising next-generation treatment or standard care for NPC. Since chemotherapy remains the primary treatment for approximately 80–90% of cancers (Chainitikun 2025), it is likely to remain in use for the foreseeable future until new effective therapies emerge. While ICIs are a promising new treatment modality, current evidences suggest that their combination with chemotherapy probably offers the most potential for improving PFS in recurrent/metastatic NPC patients.

## Data Availability

All source data for this work (or generated in this study) are available upon reasonable request.
